# Efficacy of Intra-Articular Injection of Botulinum Toxin Type A (IncobotulinumtoxinA) in Temporomandibular Joint Osteoarthritis: A Three-Arm Controlled Trial in Rats

**DOI:** 10.3390/toxins15040261

**Published:** 2023-04-01

**Authors:** Marie Béret, Florent Barry, Maria-Jose Garcia-Fernandez, Henry Chijcheapaza-Flores, Nicolas Blanchemain, Feng Chai, Romain Nicot

**Affiliations:** 1 Univ. Lille, INSERM, U1008-Advanced Drug Delivery Systems, F59000 Lille, France; 2 Univ. Lille, CHU Lille, INSERM, Department of Oral and Maxillofacial Surgery, U1008-Advanced Drug Delivery Systems, F59000 Lille, France

**Keywords:** osteoarthritis, temporomandibular joint disorders, injection intra-articular, botulinum toxins, type A

## Abstract

Temporomandibular disorders (TMD) are complex pathologies responsible for chronic orofacial pain. Intramuscular injection of botulinum toxin A (BoNT/A) has shown effectiveness in knee and shoulder osteoarthritis, as well as in some TMDs such as masticatory myofascial pain, but its use remains controversial. This study aimed to evaluate the effect of intra-articular BoNT/A injection in an animal model of temporomandibular joint osteoarthritis. A rat model of temporomandibular osteoarthritis was used to compare the effects of intra-articular injection of BoNT/A, placebo (saline), and hyaluronic acid (HA). Efficacy was compared by pain assessment (head withdrawal test), histological analysis, and imaging performed in each group at different time points until day 30. Compared with the rats receiving placebo, those receiving intra-articular BoNT/A and HA had a significant decrease in pain at day 14. The analgesic effect of BoNT/A was evident as early as day 7, and lasted until day 21. Histological and radiographic analyses showed decrease in joint inflammation in the BoNT/A and HA groups. The osteoarthritis histological score at day 30 was significantly lower in the BoNT/A group than in the other two groups (*p* = 0.016). Intra-articular injection of BoNT/A appeared to reduce pain and inflammation in experimentally induced temporomandibular osteoarthritis in rats.

## 1. Introduction

Temporomandibular disorders (TMD) are a heterogeneous group of conditions that involve the temporomandibular joint (TMJ) and associated tissues, causing chronic pain, joint noises, limitation of mandibular movement, and impaired quality of life. About 5–12% of the population in industrialized countries are estimated to suffer from TMD [1], with the most common problems being TMJ-related myalgia, arthralgia, and headache, as well as intra-articular pathologies such as disc displacement, degenerative joint disease (osteoarthrosis and osteoarthritis), and subluxation. The management of intra-articular TMD is complex and multidisciplinary. Treatment should be non-invasive in the first instance, with painkillers, physiotherapy, and stress management [2]. Oxygen–ozone therapy is the subject of some studies in the treatment of TMD and shows interesting results [3,4]. For patients not responding to noninvasive measures, pain relief may be obtained with intra-articular injection of hyaluronic acid (HA), platelet-rich plasma (PRP), or corticosteroids, but the long-term effectiveness is limited, and repeated injections increase the risk of adverse effects [5].

Chronic pain results in peripheral and central sensitization due to excess pain fiber activity, with resultant lowering of the pain threshold. Botulinum toxin type A (BoNT/A) reduces peripheral and central sensitization by decreasing the release of neuropeptides and neurotransmitters from cells or nerve endings [6,7]. Intramuscular injection of BoNT/A has been used for more than 20 years for the treatment of chronic pain and has proven efficacy in TMD with a predominantly muscular component or mixed origin [8,9].

In patients with knee or shoulder osteoarthritis resistant to systemic treatment and intra-articular HA or corticosteroid injections, intra-articular BoNT/A has shown very promising results, reducing pain and improving quality of life without causing major adverse effects [10,11,12,13,14,15,16,17,18]. In some studies, pain relief after articular BoNT/A lasted up to 8 weeks after the injection [11,13]. Furthermore, in a study of patients with non-neuropathic nociceptive knee pain, Arendt et al. [19] demonstrated a correlation between pain severity and response to BoNT/A. However, some authors are more reserved regarding the efficacy of intra-articular BoNT/A. In a study of 105 patients with knee osteoarthritis, Mendes et al. [20] showed that the pain reduction achieved with intra-articular BoNT/A was not significantly different from that achieved with intra-articular corticosteroid or saline. In a multicenter, double-blind, randomized, placebo-controlled study of 158 patients with knee osteoarthritis, McAlindon et al. [21] found no significant difference in pain reduction between patients treated with intra-articular BoNT/A versus saline. High-powered randomized controlled studies are needed to evaluate the effectiveness of intra-articular BoNT/A in the shoulder, knee, and ankle. The indications must be clearly specified, particularly the osteoarthritis stage, as BoNT/A seems to be more effective in patients with advanced disease and severe pain [22].

There is limited literature on the use of intra-articular BoNT/A in TMJ osteoarthritis (TMJOA) [8]. Two previous studies that evaluated the effect of intra-articular BoNT/A in albumin-induced TMJOA in rats reported a decrease in inflammatory mediators after the injection [23,24]. One of these studies assessed neuropeptide release and pain response (using a behavioral scale) and showed that the peripheral inhibition of release of glutamate, substance P, and calcitonin gene-related peptide (CGRP)—all neuropeptides involved in the pain signal—was responsible for the decrease in arthritis and persistent hypernociception [24]. The first clinical evaluation of intra-articular BoNT/A in humans was conducted by Batifol et al. in 2018 [25]. In this non-controlled study about patients suffering from TMD with severe chronic pain resistant to systemic treatment and intra-articular injections of HA and other drugs, intra-articular BoNT/A (30 IU administered unilaterally or bilaterally) brought about a significant reduction in temporomandibular joint pain up to 3 months after injection, but had no effect on mouth opening and joint noises. In 2022, Sari et al. [26] showed an improvement in pain relief and mouth opening with intra-articular injection of BoNT/A following arthrocentesis in patients with anterior disc displacement.

Given the poor literature but promising data on the effectiveness of BoNT/A in TMJOA, in this three-arm controlled trial we aimed to compare the effect of intra-articular BoNT/A with that of saline (placebo) and HA (as a reference molecule with proven efficacy in the treatment of articular forms of TMD) in a rat model of TMJOA induced by monosodium iodoacetate (MIA) injection.

## 2. Results

### 2.1. Clinical Observation (Body Weight Change)

**Figure 1:**
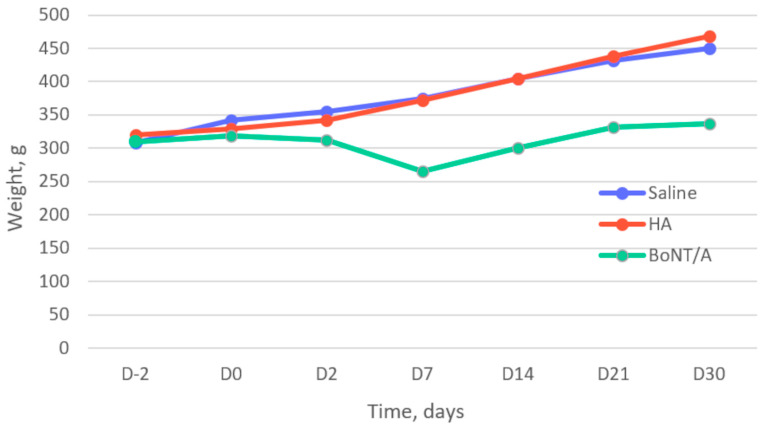
Evolution of mean weight of rats over time: before (D-2 and D0) and after (D2 to D30) intra-articular injection of placebo (saline), hyaluronic acid 1% (HA, Ostenil Mini^®^; TRB Chemedica SAS, Archamps, France), and botulinum toxin A (BoNT/A, incobotulinumtoxinA/Xeomin^®^; Merz Pharma, Frankfurt am Main, Germany).

All of the rats in the placebo and HA groups gained body weight steadily over time; in contrast, the body weight of the rats in the BoNT/A group slightly decreased initially (between day 2 and day 7) and then gradually increased until day 30 (Figure 1).

### 2.2. Nociception Assessment

**Figure 2:**
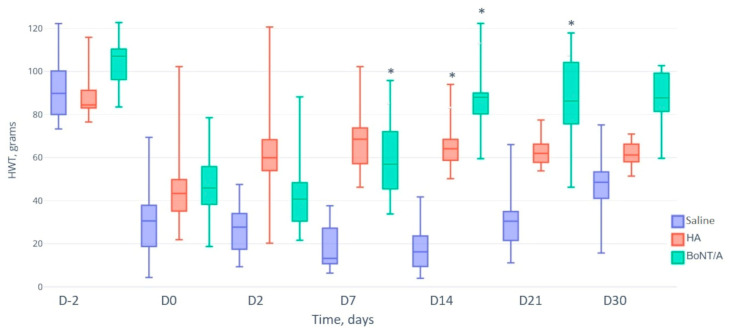
Box plot of the left temporomandibular joint head-withdrawal test values (HWT; in grams) of rats with temporomandibular joint osteoarthritis before (D-2 and D0) and after (D2 to D30) intra-articular injection of placebo (saline), hyaluronic acid 1% (HA, Ostenil Mini^®^; TRB Chemedica SAS, Archamps, France), and botulinum toxin A (BoNT/A, incobotulinumtoxinA/Xeomin^®^; Merz Pharma, Frankfurt am Main, Germany). * significant *p* value.

Table 1 and Figure 2 show the left TMJ HWT values in the three groups. In each group, the HWT values were significantly lower at day 0 (two days after induction of TMJOA) than at day −2, highlighting that the TMJOA model was well induced. In addition, at day −2 and at day 0 (two days after induction of TMJOA), the HWT values were comparable between the placebo, HA, and BoNT/A groups. At day 2, there were also no significant differences in HWT values between the three groups. At day 7, the mean HWT was significantly lower in the placebo group than in the BoNT/A group (17.56 ± 9.50 vs. 58.06 ± 18.42; *p* = 0.05). At day 14, the mean HWT was significantly lower in the placebo group than in the HA group (16.75 ± 10.29 vs. 65.88 ± 11.62; *p* = 0.028) and the BoNT/A group (16.75 ± 10.29 vs. 66.06 ± 22.53, *p* = 0.019); the difference between the HA group and the BoNT/A group was not statistically significant (*p* = 0.422). At day 21, the mean HWT was significantly lower in the placebo group than in the BoNT/A group (30.91 ± 13.64 vs. 86.01 ± 20.42; *p* = 0.048), but the differences between the placebo group and HA group or between the HA group and BoNT/A group were not statistically significant. At day 30, the mean HWT was comparable between the three groups.

### 2.3. Histological Analysis

**Figure 3:**
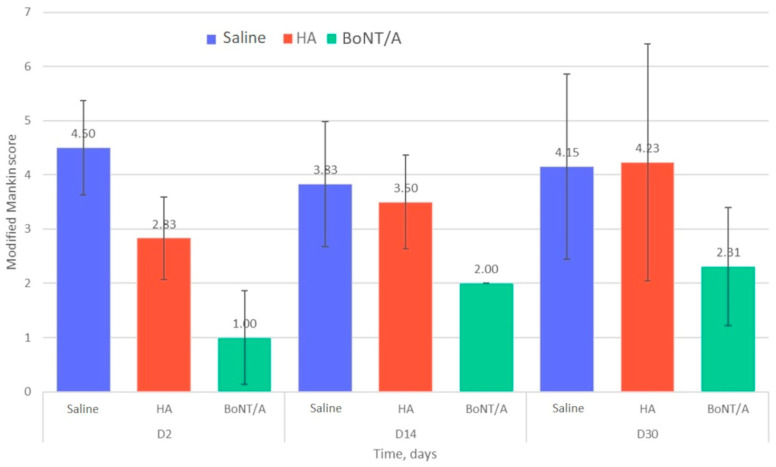
Modified Mankin histological scores of rats with temporomandibular joint osteoarthritis at day 2, day 14, and day 30 after intra-articular injection of placebo (saline), hyaluronic acid 1% (HA, Ostenil Mini^®^; TRB Chemedica SAS, Archamps, France), and botulinum toxin A (BoNT/A, incobotulinumtoxinA/Xeomin^®^; Merz Pharma, Frankfurt am Main, Germany).

Figure 3 shows the results of the histological assessment (modified Mankin histological scores) of the left TMJ (with MIA induced osteoarthritis). Changes in the profile of the histological scores over time were different in the three groups. In the placebo group, the Mankin scores were largely similar at different time points: 4.50 ± 0.87 (n = 3) at day 2, 3.83 ± 1.15 (n = 3) at day 14, and 4.15 ± 1.71 (n = 14) at day 30. In the HA group, the Mankin score increased progressively: 2.83 ± 0.76 (n = 3) at day 2, 3.50 ± 0.87 (n = 3) at day 14, and 4.00 ± 2.11 (n = 14) at day 30. Similarly, in the BoNT/A group, the Mankin score increased over time: 1.00 ± 0.87 (n = 3) at day 2, 2.00 ± 0.00 (n = 3) at day 14, and 2.31 ± 1.09 at day 30 (n = 14). The mean Mankin score at day 30 was significantly lower in the BoNT/A group than in the other two groups (*p* = 0.016).

### 2.4. ^18^FDG PET Imaging

**Figure 4:**
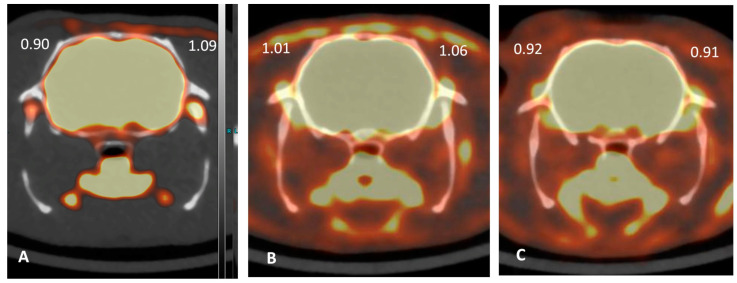
Representative ^18^FDG PET scans in a rat with temporomandibular joint osteoarthritis at day 30 after intra-articular injection of placebo (saline) (**A**), hyaluronic acid 1% (HA, Ostenil Mini^®^; TRB Chemedica SAS, Archamps, France) (**B**), and botulinum toxin A (BoNT/A, incobotulinumtoxinA/Xeomin^®^; Merz Pharma, Frankfurt am Main, Germany) (**C**).

Table 2 and Figure 4 show the standard uptake value (SUV) on ^18^FDG PET at day 30 in each group. SUV expresses the rate of ^18^FDG consumption in the area of interest, standardized by the weight of the animal and the dose injected, showing the degree of inflammation and thus osteoarthritis. The median SUV in the left TMJ was comparable between the placebo and HA groups (1.09 [0.95, 1.13] vs. 1.01 [0.86, 1.06], respectively); the median SUV in the BoNT/A group was slightly lower (0.89 [0.88, 0.91]) than the two other groups.

## 3. Discussion

This study evaluates the effect of intra-articular injection of BoNT/A (incobotulinumtoxinA/Xeomin^®^) versus intra-articular injection of saline or HA in a rat model of TMJOA. The use of the MIA-induced osteoarthritis model, based on the work of Barry et al. [27], allowed for assessment of the effect of these three substances over time. Reduction in TMJOA-related pain after intra-articular injection was similar in the BoNT/A and HA groups at day 14, with both groups having significantly better pain relief than the placebo group. Moreover, the BoNT/A group also had significantly better pain relief than the placebo group at day 7 and day 21.

### 3.1. Generalization

The results of this study are consistent with most previous studies on the subject, finding an effectiveness of BoNT/A in reducing osteoarthritis-related pain [10,11,12,13,14,15,16,17,18]. Indeed, we showed prolonged improvement in osteoarthritis-related pain from day 7 to day 21 with intra-articular injection of BoNT/A compared with intra-articular injection of the placebo (saline). Some studies also showed prolonged pain relief after intra-articular injection of BoNT/A, lasting up to 8 weeks after the injection [11,13]. Nevertheless, McAlindon et al. [21] showed contradictory results in human knee osteoarthritis, concluding to no significant difference between the intra-articular injection of BoNT/A and placebo (saline) in reducing the daily average numeric rating scale pain score over 7 days at 8 weeks. Their results are consistent with Mendes et al. [20], who found, in their randomized controlled trial, a higher short-term effectiveness of intra-articular injection of triamcinolone hexacetonide than the intra-articular injection of BoNT/A in reducing pain. It should be noted that the study involved only one dose of botulinum toxin (100 IU in a human knee), which may constitute a bias by underestimating the effectiveness of intra-articular injections of botulinum toxin. Focusing on TMJ, two previous studies on animal models of TMJOA have shown a decrease in pain mediators after intra-articular BoNT/A injection [23,24] and, in addition, Lora et al. [24] demonstrated decrease in pain in behavioral tests. Our results are in line with these previous findings in TMJOA.

Rezasoltani et al. [28] showed that intra-articular BoNT/A was more effective than HA for controlling pain and recovering function in patients in knee osteoarthritis. Conversely, Anil et al. [29] found that intra-articular stromal vascular factor, PRP, and HA were superior to BoNT/A for pain control (assessed by visual analog scale score) and functional outcomes (WOMAC score) in knee osteoarthritis. Our results showed no significant difference in pain improvement in TMJOA treated with intra-articular BoNT/A and HA; however, while pain in the BoNT/A group was significantly lower than in the placebo group at day 7, day 14, and day 21, pain in the HA group was significantly lower than in the placebo group only at day 14. Thus, our results suggest that both BoNT/A and HA can relieve TMJOA-related pain, but the effect of BoNT/A acted earlier and was more prolonged.

### 3.2. Interpretation

The observed effect of intra-articular BoNT/A on TMJOA-related pain relief is consistent with its known pharmacologic properties. Intra-articular BoNT/A inhibits the release of nociceptive neurotransmitters such as glutamate, substance P, and CGRP, leading to a reduction in pain and inflammation [24]. Glutamate is the main excitatory neurotransmitter in the nervous system of adult mammals and is involved in both pain neurotransmission and central sensitization. Glutamate release has been shown to result in inflammation, pain, and edema [7]. Meanwhile, animal models of adjuvant arthritis and of chronic inflammatory pain have shown marked upregulation of CGRP and mRNA in dorsal root ganglia neurons, as well as elevation of CGRP levels in primary afferent terminals of the spinal dorsal horn [30]. Furthermore, blocking of CGRP has been shown to block behavioral and electrophysiologic signs of enhanced pain in animals with inflammation [31]. In addition, Shi et al. [32] recently reported that the anti-inflammatory effect of BoNT/A in chronic arthritis may also be due to the inhibition of microglial cell activation and the release of microglia-derived tumor necrosis factor α (TNF-α). It is known that microglial cells are activated in chronic pain and release proinflammatory cytokines such as interleukin 6, TNFα, and interleukin 1ß, and thereby cause neuroinflammation. Moreover, P2 × 4 receptors (P2 × 4R) expressed in microglia are involved in neuropathic and inflammatory pain. All of these mechanisms may explain the pain reduction achieved by the intra-articular injection of BoNT/A.

The histological findings in this study offer further evidence in support of the efficacy of BoNT/A in treatment of TMJOA, with the mean modified Mankin score in the BoNT/A group being significantly lower than in the other two groups. The pattern of improvement of osteoarthritis over time was similar in the BoNT/A group and the HA group, but the mean Mankin scores were lower in the BoNT/A group at each time point that those in the HA group. Our findings also suggest that BoNT/A may have an early direct action on the histology, as the modified Mankin score at day 2 was 1.00 ± 0.87 in the BoNT/A group versus 4.50 ± 0.87 in the placebo group. This may be via an effect on the early inflammatory phase of osteoarthritis, with a decrease in the release of inflammatory neuropeptides and the expression of inflammatory cytokines limiting joint degradation [31,32,33]. Our results are consistent with the literature, but the mechanism of action of BoNT/A in the TMJ needs to be clarified in future studies.

The PET scan performed at day 30 in each group provided additional supportive information. SUV values were similar in the placebo and HA groups; however, they were slightly lower in the BoNT/A group. Increased ^18^FDG tracer uptake was not specific to inflammation, but it could be seen in any area with a significant glycolytic activity, for example, areas with active repair processes. This made interpretation difficult, especially in the HA group. Nevertheless, the lower bilateral SUV values in the BoNT/A group were in favor of a decrease in TMJOA at day 30 and corroborated the clinical and histological findings.

### 3.3. Limitations of the Study

This study has several limitations. The first was the choice of HA as the reference intra-articular treatment for TMJOA. We chose HA because it is currently the most widely used intra-articular treatment for TMJOA because of its viscosupplementation properties [5]. Other injectable substances such as corticosteroids and PRP are also used. Intra-articular corticosteroids, alone or in combination with other drugs, have not shown better results than intra-articular HA and, moreover, are associated with a risk of condylar resorption [5]. Several studies have shown good results with intra-articular PRP in TMJOA, but the manufacturing protocol is not standardized, and time and special equipment are required to obtain PRP [5,29,34,35,36]. Second, the weight of the rats in the BoNT/A group initially decreased due to feeding difficulties, probably due to muscle weakness caused by the diffusion of BoNT/A into the masticatory muscles. Change from a hard to a soft diet allowed the rats to eat normally and regain weight. This change in the weight and diet of the rats may have induced stress and behavioral changes, which may have resulted in an underestimation of the pain improvement in the BoNT/A group. In addition, the volume and concentration of the injected BoNT/A was based on the articles by Lora et al. [23,24], and recent studies in humans on the use of high doses of toxin showed a rare occurrence of adverse effects [37,38]. Third, the study sample size of the study was calculated for the statistical analysis of nociception; this sample may have been too small for the statistical analysis of histological and PET imaging data.

## 4. Conclusions

Intra-articular injection of BoNT/A (incobotulinumtoxinA/Xeomin^®^) appears to be effective for reducing pain in experimentally induced TMJOA in rats. Histological and PET imaging findings support these results. The mean Mankin osteoarthritis histological score at day 30 was significantly lower in the BoNT/A group than in the other two groups.

More high-powered preclinical and clinical studies are needed to determine the place of intra-articular injection of BoNT/A for the treatment of temporomandibular joint osteoarthritis and to draw a firm conclusion.

## 5. Material and Methods

### 5.1. Animals

Sixty male Wistar rats (6-weeks-old; weight of 250–300 g) were included in this study. The rats were housed in individual cages in a temperature-controlled room (22 °C ± 1 °C) with a 12-h dark–light cycle and allowed for free access to food and water. Manipulations started after ten days of quarantine.

All of the procedures in this study were approved by Ministère de l’enseignement supérieur, de la recherche et de l’innovation (APAFIS#25897, 29/10/2020).

### 5.2. Induction of Temporomandibular Joint Osteoarthritis and Injection Protocol

The animals were anesthetized by the inhalation of isoflurane mixed with pure oxygen (flow rate: 1.5 L/min) for 2–3 min in a Plexiglas^®^ chamber. TMJOA was induced in the left TMJ of all rats by intra-articular injection of monosodium iodoacetate (MIA) into the upper compartment in normal saline (0.5 mg/50 μL of saline; Sigma, Saint Louis, MI, USA) [27,39]. The injection protocol was based on the work of Fuentes et al. [40]. Two days after MIA injection, the rats were anesthetized by the same technique and then randomly divided into three groups: 20 rats (the BoNT/A group) received intra-articular injection of 2.5 UI/50 µL BoNT/A (incobotulinumtoxinA; Xeomin^®^; Merz Pharma, Frankfurt am Main, Germany) in the left and right joint of each rat; 20 rats (the HA group) received intra-articular injection of 50 µL of 1% HA (Ostenil Mini^®^; TRB Chemedica SAS, Archamps, France) in the left and right joint of each rat; and 20 rats (the placebo group or saline group) received intra-articular injection of 50 µL of 0.9% saline in the left and right joint of each rat. Both Xeomin^®^ and Ostenil^®^ were selected because they are used in clinical practice. In addition, Ostenil^®^ has European certification for use in small joints, including TMJ. Neither Merz Pharma or TRB Chemedica were sponsors of the study.

### 5.3. Nociception Assessment

**Figure 5:**
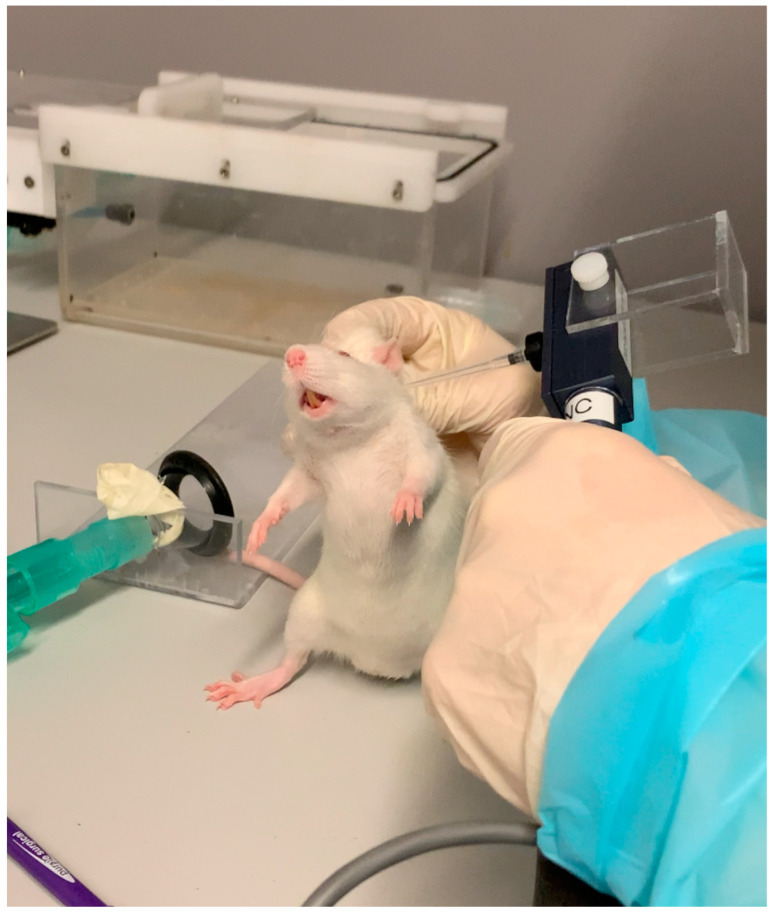
Head withdrawal test method: gradually increasing pressure was applied using a Von Frey esthesiometer to the temporomandibular joint area until the animal withdrew its head, vocalized, or both, and the lowest value of pressure (in grams) that induced a response was recorded. The gesture was performed on each temporomandibular joint of each rat.

The head-withdrawal test (HWT) was used to assess pain. According to the systematic review by Nicot et al. [39], long-term pain related to TMJOA has mostly been assessed by measuring the threshold pressure value (in grams) that triggers a pain response. In this study, a Von Frey aesthesiometer (Figure 5) was applied with gradually increasing pressure to the rat TMJ till head withdrawal, vocalization, or both occurred, indicating nociception; the threshold value was defined as the lowest pressure that induced the response. After each measurement, the rats were weighed (in grams) to monitor their general wellbeing before being returned to their cages.

### 5.4. Histological Analysis

In each group, randomly selected animals were humanely killed at day 2 (n = 3), day 14 (n = 3), and day 30 (n = 14) by intracardiac injection of 0.2 mL of T61 under isoflurane anesthesia and then immediately stored at −20 °C for at least 48 h. Then, clean-cut samples of approximately 5 mm thickness were obtained from the TMJ area. The samples were first placed in cassettes and fixed in 4% formaldehyde solution for 24 h, and then decalcified by immersion in 15% ethylenediaminetetraacetic acid (EDTA; pH 7.2) solution for 5 days. The prepared samples were stored in 70% ethanol solution at 4 °C until histological processing (paraffin embedding, cutting, and staining) and analysis.

Briefly, the sections were first stained with hematoxylin and eosin (HE) staining to select the slides of interest. The selected slides were thus stained with toluidine blue stain (TB) and examined under the microscope for determining the histological osteoarthritis score using the modified Mankin scale (Table 3). The higher the final score on the Mankin scale, the more advanced the TMJOA stage [41,42].

### 5.5. Positron Emission Tomography (PET) Imaging

PET with 2-deoxy-2-[18F] fluoro-D-glucose (^18^FDG) was carried out for monitoring the stage of inflammation. ^18^FDG radiotracer was used to visualize the areas of high glucose consumption, caused in this case by synovitis and TMJOA bone lesions [43,44]. Imaging was performed at day 30 on five randomly selected rats in each group. The rats were fasted the day before the examination. Intravenous administration of the ^18^FDG radiotracer (30–35 MBq) and image acquisition were carried out under general anesthesia. Manipulations were performed in compliance with the rules of human radioprotection [45]. The animals were isolated the first 24 h after radiotracer injection.

### 5.6. Full Protocol Frame of the Study

**Figure 6:**
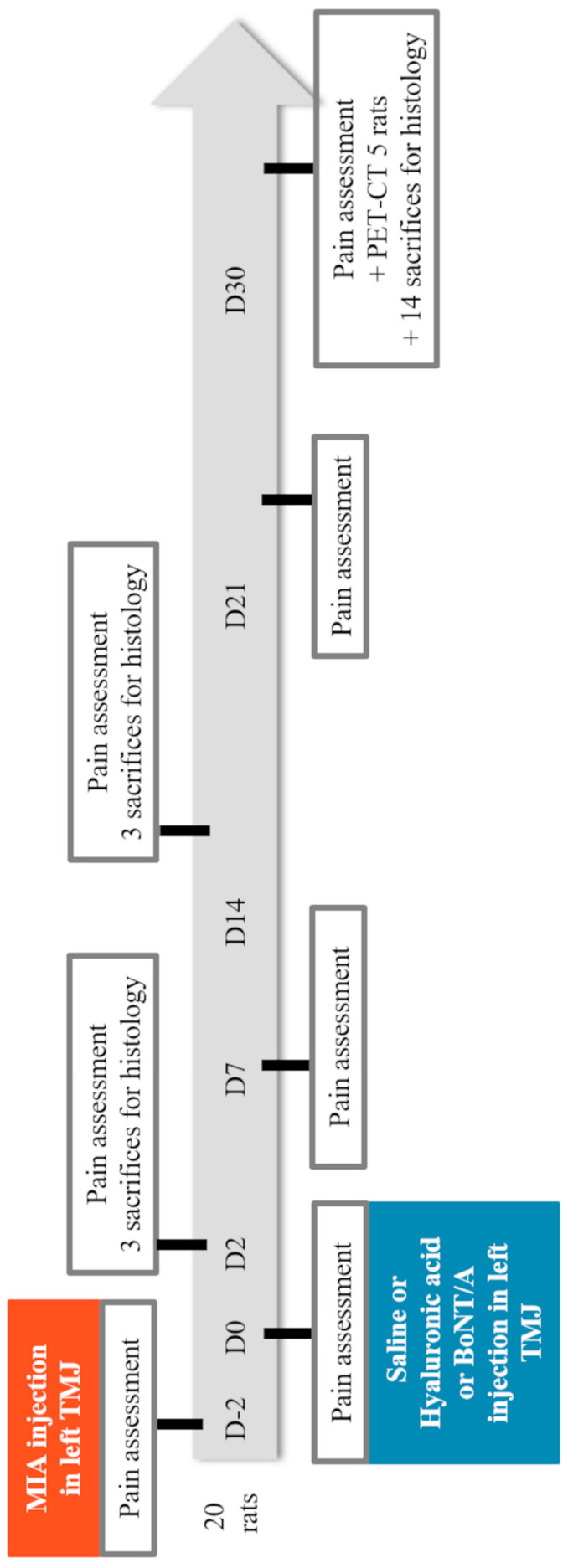
Full experimental protocol from temporomandibular joint osteoarthritis induction (day −2) to day 30 after therapeutic joint injection.

Figure 6 summarizes the basic frame structure of the full protocol of analysis described below: from TMJOA induction (day −2) to day 30 after therapeutic joint injection.

### 5.7. Statistical Analysis

The sample size was calculated for three-group one-way analysis of variance (ANOVA) using G*Power 3.1 (Heinrich-Heine-Universität Düsseldorf, Düsseldorf, Germany), assuming α  = 0.05, β  =  0.2, standard deviation (SD)  =  10, and effect size  =  0.42. The calculated sample size was 19 per group. The final sample size was set at 20 rats per group, keeping in mind potential animal losses and the 3R’s rule for experimental procedures in animals [46]. Quantitative variables were expressed as means (±standard deviation) or medians (interquartile range; Q1, Q3), depending on the normality of the distribution. The normality of distributions was assessed using histograms and the Shapiro–Wilk test. Categorical variables were expressed as numbers (percentage). The mean weights and HWT values on day −2 and day 0 were first compared to check the comparability of the three groups. One-way ANOVA was used to compare the HWT values in the three groups. Levene’s test was used to test the homogeneity assumption required by ANOVA. Multiple comparisons within the experimental groups were performed using Tukey’s test. One-way ANOVA followed by Dunnett’s test was used to compare the placebo group with the experimental groups. Kruskal–Wallis test was used to compare the left TMJ Mankin score at day 30 because the assumptions of one-way ANOVA were not met. All of the statistical analyses were performed using XLSTAT 2022.5.1 (Addinsoft, New York, NY, USA). Statistical significance was at *p* < 0.05.

## Figures and Tables

**Table 1:** Left temporomandibular joint head-withdrawal test values (in grams) of rats with temporomandibular joint osteoarthritis at different time points: before (D-2 and D0) and after (D2 to D30) intra-articular injection of placebo (saline), hyaluronic acid 1% (HA, Ostenil Mini^®^; TRB Chemedica SAS, Archamps, France), and botulinum toxin A (BoNT/A, incobotulinumtoxinA/Xeomin^®^; Merz Pharma, Frankfurt am Main, Germany). * significant *p* value.

Left HWT, Grams Mean ± SD	D-2	D0	D2	D7	D14	D21	D30
**Saline**	91.99 ± 15.03	29.81 ± 15.48	26.71 ± 9.96	17.56 ± 9.50	16.75 ± 10.29	30.91 ± 13.64	47.54 ± 14.19
**HA**	88.02 ± 9.59	44.85 ± 16.68	62.96 ± 21.69	67.75 ± 15.04	65.88 ± 11.62	63.03 ± 7.31	61.47 ± 6.15
***p***	0.99	0.41	0.20	0.17	0.03 *	0.16	0.52
**Saline**	91.99 ± 15.03	29.81 ± 15.48	26.71 ± 9.96	17.56 ± 9.50	16.75 ± 10.29	30.91 ± 13.64	47.54 ± 14.19
**BoNT/A**	103.87 ± 11.63	45.92 ± 15.39	43.73 ± 17.73	58.06 ± 18.42	66.06 ± 22.53	86.01 ± 20.42	87.48 ± 11.92
***p***	0.90	0.47	0.45	0.05 *	0.02 *	0.05 *	0.22
**HA**	88.02 ± 9.59	44.85 ± 16.68	62.96 ± 21.69	67.75 ± 15.04	65.88 ± 11.62	63.03 ± 7.31	61.47 ± 6.15
**BoNT/A**	103.87 ± 11.63	45.92 ± 15.39	43.73 ± 17.73	58.06 ± 18.42	66.06 ± 22.53	86.01 ± 20.42	87.48 ± 11.92
***p***	0.91	0.83	0.99	0.70	0.42	0.28	0.46

**Table 2:** Standard uptake value measured on ^18^FDG positron emission tomography scans performed at day 30 after intra-articular injection of placebo (saline), hyaluronic acid 1% (HA, Ostenil Mini^®^; TRB Chemedica SAS, Archamps, France), and botulinum toxin A (BoNT/A, incobotulinumtoxinA/Xeomin^®^; Merz Pharma, Frankfurt am Main, Germany).

	SUV–Mdn (Q1;Q3)	
	Left	Right
Saline (n = 5)	1.09 (0.95;1.13)	0.9 (0.79;0,90)
HA (n = 5)	1.01 (0.86;1.06)	1.01 (0.89;1.08)
BoNT/A (n = 5)	0.89 (0.88;0.91)	0.92 (0.83;0.93)

**Table 3:** Modified Mankin scale for histological scoring of temporomandibular joint osteoarthritis.

Structure	
Normal	0
Surface irregularities	1
Pannus	2
Cleft to transitional zone	3
Cleft to radial zone	4
Cleft to calcified zone	5
Complete disorganization	6
**Tidemark integrity**	
Intact	0
Crossed by blood vessels	1
**Proteoglycan staining**	
Normal	0
Slight reduction	1
Moderate reduction	2
Severe reduction	3
No dye noted	4
**Cellularity**	
Normal	0
Diffuse hypercellularity	1
Cloning	2
Hypocellularity	3

## Data Availability

Data available on request due to restrictions eg privacy or ethical.
